# Redesigning an Electronic Health Record in Argentina to Improve Comprehensive Health Care and Clinical Research

**DOI:** 10.3233/SHTI220234

**Published:** 2022-06-06

**Authors:** Liliana Giraldo, Valeria Fink, Florencia Cahn, Betiana Caceres, Omar Sued, Stephany Duda, Carina Cesar

**Affiliations:** a Fundación Huésped, Buenos Aires, Argentina; b Centro Médico Huésped, Buenos Aires, Argentina; c Department of Biomedical Informatics, Vanderbilt University School of Medicine, Nashville, TN, USA

**Keywords:** Electronic Health Records, Communicable Diseases, Argentina

## Abstract

Updating electronic health record systems to meet new clinic needs and government regulations presents an ongoing challenge for health care organizations. To redesign an existing system for two HIV clinics in Argentina, we employed a three-phase approach of exploration, participatory design, and prototyping. The process and resulting architecture of the HIV-centered “RedClin” electronic health record may inform electronic health records at other clinics in Latin America and worldwide.

## Introduction

Electronic health record systems (EHRs) can improve clinical data collection and deliver structured data to develop clinical research [1], but redesigning such software to accommodate new regulations and specialized environments is difficult [2]. General challenges exist in identifying health providers’ needs, transforming workflow processes, and encouraging users to adapt to change [3–5]. EHR interfaces should support robust data collection and guide clinicians in avoiding errors due to missed interventions or inadequate visit documentation [3]. Our collaborating clinics in Argentina had experience with existing, locally developed software, but had to meet workflow requirements and new government regulations for EHRs before the system could be installed at new clinics. This paper ‘s objective is to describe the process of redesigning the user interface and architecture of an existing EHR with specific requirements for Infectious Diseases, HIV care, and clinical research.

## Methods

Our EHR development took place at Fundación Huésped, a regional Argentine organization that has been working in public health since 1989, providing clinical care for and conducting research on HIV/AIDS, viral hepatitis, vaccine-preventable diseases and other transmissible diseases, as well as sexual and reproductive health. In 2011, Fundacion Huésped developed an HIV care-centric EHR used in the Infectious Diseases division of a public hospital in Buenos Aires. In 2017, we identified a need for an EHR at Fundación Huésped and a second clinic, Centro Medico Huésped. Based on provider and IT experience with the existing hospital system, a decision was made to redesign the EHR for clinic use. The aims of that process were to enhance the EHR to include additional features in support of research including integration of formal data standards for field coding, data integrity checks, and standardized data export formats. At the same time, we aimed to package the EHR for opensource sharing by modularizing and generalizing core software features, implementing a cloud-based system for data security and future scalability, and developing a framework for sharing the EHR with other HIV clinics in Argentina.

Our work proceeded in three phases:

### Phase 1: Exploration

First, we analyzed the strengths and weaknesses of the existing EHR with the objective of improving usability, collecting structured data according to international standards, and porting the system to the cloud. We interviewed five health care providers to collect their EHR feedback and assess their needs. We also conducted a work-sampling through contextual observation of health care providers at Fundacion Huésped and Centro Medico Huésped to analyze how the professionals worked during their working day, taking into account the time allocated to the recording of clinical information. The collective input informed development of low-fidelity prototypes [6]. The IT team also identified necessary modifications to the design and system architecture to support cloud-based installation.

### Phase 2: Participatory design

The participatory design phase was conducted based on the previous phase and low-fidelity prototypes. Health care providers gave their opinions after interacting with the first prototypes and the development was oriented by their inputs. There were two cycles of prototyping and testing. The emphasis during this phase was qualitative, looking for domain saturation. Opinions and thoughts of health care providers were obtained and recorded using the Think Aloud technique.

### Phase 3: Prototype design

Appropriate adjustments were made to reach a minimum viable product (MVP) for the revised EHR. Infrastructure requirements were also defined and implemented.

## Results

Several features were carried over from the original EHR. However, the multi-phase redesign process identified new design and development requirements that were implemented in the final prototype. The central module of our new “RedClin” EHR housed the patient index; functions for search and creation of new patients were available on the initial screen. A minimum dataset was established for patient search (e.g., social or legal name, surname, ID number). The definition of the minimum dataset was based on guidelines from the Argentina National Ministry of Health on unique identification of people in the health system [7]. This approach reduced errors in patient identification and prevented the creation of duplicate patients. It also enabled interoperability with other information systems using on the same identification standards.

Additional modules were designed to record health data during the clinical visit: Clinic Notes (narrative of the current visit and past medical history), Problems (active and resolved problems, family history), Medication List (active and past antiretrovirals, opportunistic infection prophylaxis, and other prescribed drugs), Laboratory Results (imported through integration with laboratory systems as well as manual entry, with graphical trends over time), Other Procedures and Tests (e.g., endoscopy results, cardiac stress tests, brain MRI), Immunizations (ordering and administration of vaccines). An EHR Summary module and HIV Summary module provided targeted reports for clinician use.

Throughout the implementation, we used standardized terminologies to record structured data. Diagnoses and laboratory values were coded according to the International Statistical Classification of Diseases and Related Health Problems (ICD) and Logical Observations Identifiers, Names, Codes (LOINC) terminologies. An additional feature supported participation in research collaborations; the data could be exported using the common data model for the International epidemiology Databases to Evaluate AIDS (IeDEA), formatted for merging with other participating research sites for multiregional collaborations.

The improved EHR is a cloud-based platform accessible from any place with an Internet connection and a web browser. The solution was migrated from an on-premises infrastructure to virtual servers in a cloud platform hosted in Argentina, as required by national data protection regulations. The web application and the architecture are supported by a collaboration among the development and infrastructure teams in different virtual environments (DEV, UAT and PROD). The source code is shared among the team members by using a private GitHub repository and implemented with good programming practices.

## Discussion

Information systems in the healthcare environment allow for systematic processing of data and contribute meaningfully to the health organization’s progress. This multi-phase process allowed us to design and build a custom EHR that met the data collection needs of clinical, administrative, and research staff. We found the redesigned EHR enhanced care continuity due to better team communication and ubiquitous access to clinical information. Electronic prescribing and typed clinic notes have improved the interpretability of medical records. Furthermore, the research-driven data export options have simplified the two clinics’ abilities to contribute data to epidemiological cohort studies by conducting automatic quality checks and formatting data for scientific exchange.

Some clinics with existing EHR solutions may choose to replace their systems with vendor EHR software rather than modernize the existing EHR. However, many of these EHR solutions are complex and require advanced technical expertise to install and customize. We have demonstrated a feasible approach to redesign a domain-specific EHR and successfully implement it in two clinics. We plan to package and release the RedClin EHR so other HIV and infectious diseases clinics in Argentina and other Spanish-speaking settings can benefit from this software as well as our EHR redesign methodology.

## Conclusions

Outdated EHRs can be successfully redesigned using an iterative, user-engaged process. The RedClin system is contributing to comprehensive health care, data collection, and clinical research at HIV clinics in Buenos Aires.
